# Supplementary material for: Influence of Ni and Sn Perovskite NiSn(OH)6 Nanoparticles on Energy Storage Applications
Source: Nanomaterials (Basel). 2023 Apr 30;13(9):1523. doi: 10.3390/nano13091523 (PMC10179963; doi:10.3390/nano13091523)
Supplement: Supplementary file 1 [file nanomaterials-13-01523-s001.zip › nanomaterials-2342575-Supplementary.pdf]

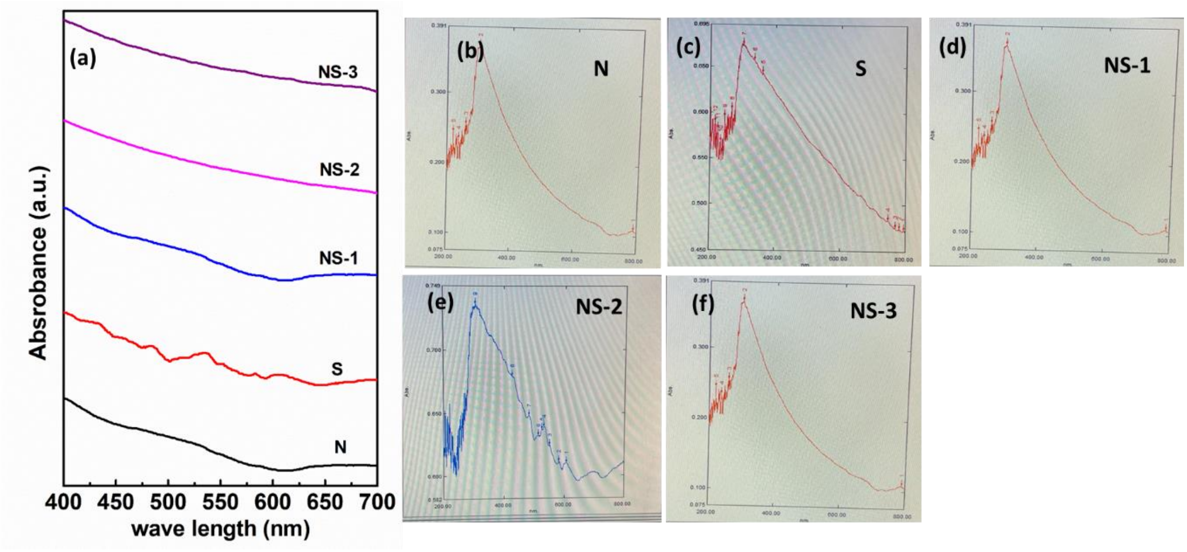

Figure S1. UV spectra of prepared samples.

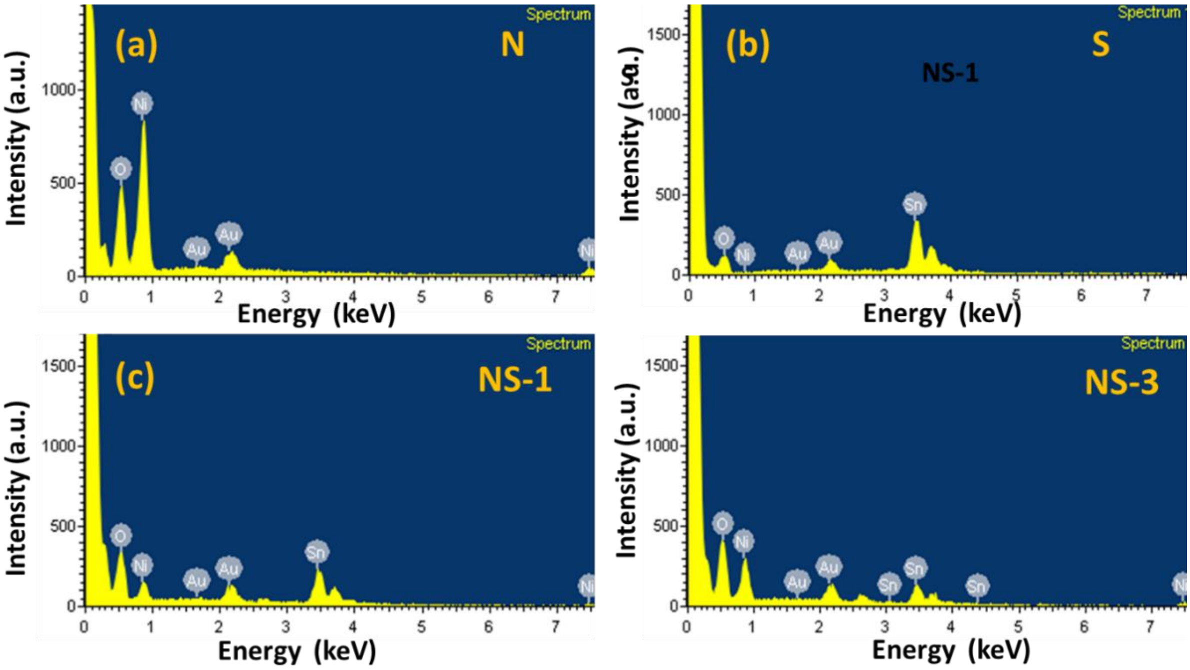

Figure S2. The EDAX spectra of (a) N, (b) S, (c) NS-1 and (d) NS-3 samples.

Table S1. Specific capacitance of $NiSn(OH)_6$ hydroxide nanomaterials.

Current Density(A/g)	Specific Capacitance (F/g)				
	**N**	**S**	**NS-1**	**NS-2**	**NS-3**
1	380	323	509	607	565
2	276	219	328	450	350
3	225	181	272	410	290
4	190	123	210	388	225
5	160	105	170	338	188
